# Iliopsoas tendonitis following total hip replacement in highly dysplastic hips: a retrospective study

**DOI:** 10.1186/s13018-019-1176-z

**Published:** 2019-05-22

**Authors:** Junfeng Zhu, Yang Li, Kangming Chen, Fei Xiao, Chao Shen, Jianping Peng, Xiaodong Chen

**Affiliations:** 1 Department of Orthopaedics, Xinhua Hospital Affiliated to Shanghai Jiaotong University School of Medicine, Building 8, No.1665, Kongjiang Road, Shanghai, 200092 China; 2 Huashan Hospital, Fudan University, Shanghai, China

**Keywords:** Iliopsoas tendonitis, Hip dysplasia, Total hip arthroplasty, Hip instability

## Abstract

**Background:**

As a recognized cause of groin pain following total hip arthroplasty, iliopsoas tendonitis probably results from different factors. Given the anatomic disadvantage, dysplastic hips theoretically make acetabular component relatively retroverted or oversized, screws implanted frequently, and iliopsoas tendonitis more likely. However, the prevalence and mechanism of iliopsoas tendonitis following total hip replacement in dysplastic hips are not fully understood.

**Methods:**

One hundred and thirty-three total hip arthroplasties for Crowe type 2 to 4 dysplastic hips were compared with 126 total hip arthroplasties for hips without dysplasia in this study. Preoperative patient demographic data were well matched between the groups. Clinical and radiographic evaluations were performed.

**Results:**

A significantly higher frequency of protruded screws (24.8% vs 0), anterior overhang of acetabular components (30.8% vs 4.0%), and increased leg lengthening (3.6 [2.0–6.8] vs 0.5 [0–1.8]) was found in the dysplastic group (all *p* values < 0.05). However, the femoral offset and inclination and anteversion of acetabular components between the groups did not differ significantly. No difference in the prevalence of iliopsoas tendonitis was found between the groups. A new cause of iliopsoas tendonitis following total hip arthroplasty was detected in the dysplastic group. The iliopsoas tendonitis was irritated by an instable artificial femoral head.

**Conclusions:**

The dysplastic hips did not present a higher incidence of postoperative iliopsoas tendonitis in this study. Iliopsoas tendonitis could be somewhat prevented by smaller size of acetabular components and soft tissue release in dysplastic hips, but irritated by an instable artificial femoral head.

## Background

Besides infection and aseptic loosening, iliopsoas tendonitis is another reason for painful hip replacement. The reported prevalence of iliopsoas tendonitis is up to 4.3% in patients with total hip arthroplasty [1, 2]. However, the mechanism is still not fully understood. Impingement against a relatively retroverted or oversized acetabular component has been frequently detected as a common cause of iliopsoas tendonitis [3, 4]. Furthermore, protruded screws, increased femoral offset or leg length, overhang of a collar femoral prosthesis, presence of a larger-diameter femoral ball, acetabular cage or reinforcement ring, and recurrent hematomas inside the iliopsoas muscle are related to iliopsoas tendonitis [5–10]. When conservative treatments failed in treating replaced hips with iliopsoas tendonitis, iliopsoas tendon release or acetabular component revision was usually suggested.

Total hip arthroplasty in dysplastic hips presents specific difficulties including reduced acetabulum depth, anterolateral and superior acetabular bone deficiency, leg length discrepancy, and muscular contractures. These features are relatively severe in Crowe type 2 to 4 [11, 12] dysplastic hips. Theoretically, these features make the acetabular component relatively retroverted or anteriorly oversized, screws implanted frequently, and iliopsoas tendonitis more likely. Recently, Jacobsen et al. reported a high prevalence of iliopsoas-related pain prior to scheduled periacetabular osteotomy for Crowe type 1 dysplastic hips [13]. However, few studies have reported on the prevalence of iliopsoas tendonitis or iliopsoas-related pain following total hip replacement in highly dysplastic hips. Given the anatomic disadvantage of dysplastic hips, we addressed the following questions: (1) How to define the risk of anteriorly oversized acetabular components, protruded screws, and increased leg length following total hip replacement in highly (Crowe type 2 to 4) dysplastic hips? (2) Do highly dysplastic hips present a higher incidence of postoperative iliopsoas tendonitis? (3) Is there any other factors related to postoperative iliopsoas tendonitis?

## Methods

### Patients

A consecutive series of Crowe type 2 to 4 [11] dysplastic hip was retrospectively identified at our institution between March 2009 and December 2016. Patients with preoperative iliopsoas-related pain were excluded based on physical examination [13]. A total of 133 hips in 118 patients were identified and investigated based on the ethical standards of the committee on human experimentation in our institution. A well-matched control group of 126 primary total hip arthroplasties performed during the same period in 115 patients without hip dysplasia was chosen from the medical records in our institution. The cause of hip replacement in control group included primary osteoarthritis, femoral head necrosis, ankylosing spondylitis, osteoarthritis following slipped capital femoral epiphysis, and hip chondrolysis. Patient demographic data including age, gender, body mass index, previous surgical procedures, and follow-up period are shown in Table 1. None of them differed significantly between the groups.

**Table 1:** Patient’s demographic data

Parameters	DDH group (*n* = 133)	Control group (*n* = 126)	*p* value
Age (years old)	36.4 ± 11.6 (21–77)^d^	38.5 ± 9.8 (17–75)^d^	0.106^a^
Gender (male/female)	8/125	7/119	0.874^b^
Body mass index (kg/m^2^)	23.1 ± 2.5 (18.6––28.4)^d^	23.4 ± 2.5 (19.5–30.2)^d^	0.325^a^
Previous surgical procedures	17 (12.8%) Open reduction (7) Pelvic osteotomy (5) Proximal femoral osteotomy (4) Hip arthrocopy (1)	15 (11.9%) Core decompression (5) Bone graft (5) Internal fixation (3) Osteochondral plasty (2)	0.830^b^
Follow-up period (month)	45.9 ± 18.9 (18–92)^d^	44.8 ± 17.7 (18–96)^d^	0.626^a^

^a^Student’s *t* test

^b^Pearson’s chi-square test

^d^Values are expressed as mean ± standard deviation with range in parentheses

### Surgical technique

A posterolateral approach was performed by one senior surgeon (X. Chen) to expose the hip joint and proximal femur in all cases. After capsulotomy, the true acetabulum of a dysplastic hip was identified by removing the soft tissue within it. A cementless acetabular component (Pinnacle, DePuy, Warsaw, IN, USA, or Trilogy, Zimmer, Warsaw, IN, USA) was placed at the level of the original acetabulum or a slightly elevated position without lateralization. If the original acetabulum bone stock was severely deficient, bulk autografts from the resected femoral head were fixed with screws to provide acetabular component coverage. The acetabular component was fixed with screws and an intended anteversion of 25°–30° in all cases. After the implantation of the acetabular component, the femur cavity was prepared. A cementless femoral stem (Corail, DePuy, Warsaw, IN, USA; Summit, DePuy, Warsaw, IN, USA; or Wagner Cone, Zimmer, Warsaw, IN, USA) was selected based on the preoperative templating. In some of the dislocated hips, a subtrochanteric shortening osteotomy was performed as described in our previous report [12]. Prophylactic wire fixation was used before the insertion of the uncemented Wagner Cone Stem (Zimmer, Warsaw, IN, USA) to prevent iatrogenic splitting of the femur. For Crowe type 4 dysplastic hips, the iliopsoas tendon was released if the femoral head could not be reduced, at the insertion of lesser trochanter. The muscle-tendon unit of iliopsoas was not completely interrupted. The tendon unit was cut off using an electrotome, whereas the muscle unit remained intact. The wear bearing materials of this cohort consisted of ceramic-on-ceramic in 128 hips, ceramic-on-poly in 2, and metal-on-poly in 3 hips. The patients were hospitalized for an average of 7.4 days (range 5–11 days). If a subtrochanteric shortening osteotomy was performed, partial weight bearing was encouraged at 3–6 weeks postoperatively. Twelve weeks later, full weight bearing was usually allowed if osseous healing was positive at the osteotomy sites.

### Clinical and radiographic evaluation

Clinical results were evaluated at 3 months, 6 months, and 1 year postoperatively, and then yearly until the last follow-up. The diagnostic criteria for iliopsoas tendonitis following total hip replacement were defined as follows: (1) pronounced hip groin pain while the operated hip was lifting to a bed or into a ca; (2) physical examination-indicated palpatory pain was related to the movement of iliopsoas tendon [13], especially resisted hip flexion; (3) the groin pain immediately relieved after a peritendinous injection with corticosteroid and local anesthetics[14]; (4) infection, loosening, and osseous nonunion were excluded; and (5) examination of the spine and abdomen was negative.

Preoperative leg length discrepancy (LLD) was measured as we previously described. LLD indicated the length discrepancy of lower extremities which was measured with a tape by measuring the distance between the anterior superior iliac spine and the medial malleolus [12]. Anteroposterior (AP) radiographs were made at follow-up visits. Radiographic evaluations were performed by one surgeon (J. Zhu) including inclination of acetabular components, anteversion of acetabular components, femoral offset, protruded screw, leg lengthening, loosening signs, and bone union of the femoral osteotomy. Inclination of the acetabular component is the angle between a line through the long axis of its ellipse and the inter-teardrop line on AP radiographs. Anteversion of the acetabular component was measured on AP radiographs by using Liaw’s method [15]. Anteversion = sin^−1^ tan *β*. *β* was the angle formed by the long axis of the component and the line connecting the top point of the ellipse and the endpoint of the long axis [15]. The femoral offset was defined by measuring the distance on AP radiographs between the femoral axis and the femoral head center [12]. The protruded screw was defined as a screw perforating the inner acetabular cortex on radiographs. Protrusion was identified as soon as the inner acetabular cortex was perforated by the screw head [8]. The amount of radiographic leg lengthening was measured by subtracting the amount of intraoperative femoral resection from the distance between the top of the greater trochanter preoperatively and postoperatively on radiographs [16]. The anterior overhang of the acetabular component was detected on surgery records or computed tomographic (CT) scan postoperatively. The stability of the hip components was assessed radiographically as previously described [17]. Provided consolidation presented across the osteotomy sites, osseous healing was determined.

### Statistical analysis

Statistical analyses were conducted using SPSS statistics 19 (IBM, Armonk, NY). The preoperative LLD, femoral offset, inclination and anteversion of acetabular components, acetabular component size, leg lengthening, prevalence of protruded screw, anterior overhang of acetabular component, and postoperative iliopsoas tendonitis in the dysplastic group were compared with those of the control group by using Student’s *t* test and Pearson’s chi-square test. Significance was determined at a *p* value of < 0.05.

## Results

The results were shown in Table 2. Preoperative LLD was higher (3.7 [2.0–7.0] vs 0.5 [0–1.6]) in the dysplastic group. The femoral offset and inclination and anteversion of acetabular components between the groups did not differ significantly. A significantly higher frequency of protruded screws (24.8% vs 0), anterior overhang of acetabular components (30.8% vs 4.0%), and increased leg lengthening (3.6 [2.0–6.8] vs 0.5 [0–1.8]) was found in the dysplastic group (all *p* values < 0.05). However, no difference in the prevalence of iliopsoas tendonitis was found between the groups.

**Table 2:** Postoperative clinical and radiographic evaluation

Parameters	DDH group (*n* = 133)	Control group (*n* = 126)	*p* value
Acetabular component size (mm)	44.6 ± 2.0 (38–48)^d^	47.9 ± 2.8 (44–56)^d^	0.000^a^
Protruded screw	33 (24.8%)	0	0.000^b^
Anterior overhang of acetabular components	41 (30.8%)	5 (4.0%)	0.000^b^
Inclination of acetabular components(°)	42.0 ± 2.5 (36.0–47.4)^d^	41.6 ± 2.7 (35.1–50.3)^d^	0.177^a^
Anteversion of acetabular components(°)	26.8 ± 2.6 (20.5–43.0)^d^	26.6 ± 3.5 (20.2–35.1)^d^	0.568^a^
Femoral off-set (cm)	3.4 ± 0.3 (2.5–4.1)^d^	3.5 ± 0.4 (2.7–4.2)^d^	0.128^a^
Preoperative leg length discrepancy (cm)	3.7 ± 1.0 (2.0–7.0)^d^	0.5 ± 0.3 (0–1.6)^d^	0.000^a^
Leg lengthening (cm)	3.6 ± 0.8 (2.0–6.8)^d^	0.5 ± 0.2 (0–1.8)^d^	0.000^a^
Iliopsoas tendon release	56 (42.1%)	0	0.000^b^
Iliopsoas tendonitis	2 (1.5%) 2 (2.6%^e^)	1 (0.8%) 1 (0.8%)	1.000^c^ 664^c^

^a^Student’s *t* test

^b^Pearson’s chi-square test

^c^Pearson’s chi-square test with continuity correction

^d^Values are expressed as mean ± standard deviation with range in parentheses

^e^Incidence of iliopsoas tendonitis in DDH group excluding the cases with iliopsoas tendon release in surgerie

No hip failed in the control group. The anterior overhang of the acetabular component was recorded in five cases. Iliopsoas tendonitis was only detected with the maximal size of acetabular component (56 mm). At 6 months postoperatively, groin pain in this case was successfully treated with peritendinous injection of a local anesthetic and corticosteroid.

In the dysplastic group, groin pain appeared postoperatively in three cases. One of the three hips was failed for posterior hip dislocation and femoral component loosening at 6-month follow-up. Iliopsoas tendonitis was diagnosed in the other two hips at 5 months postoperatively. The leg lengthening was 4.5 cm and 2 cm, respectively. The injection successfully relieved groin pain in the 4.5-cm lengthened hip but failed in the other to present lasting relief.

**Fig. 1:**
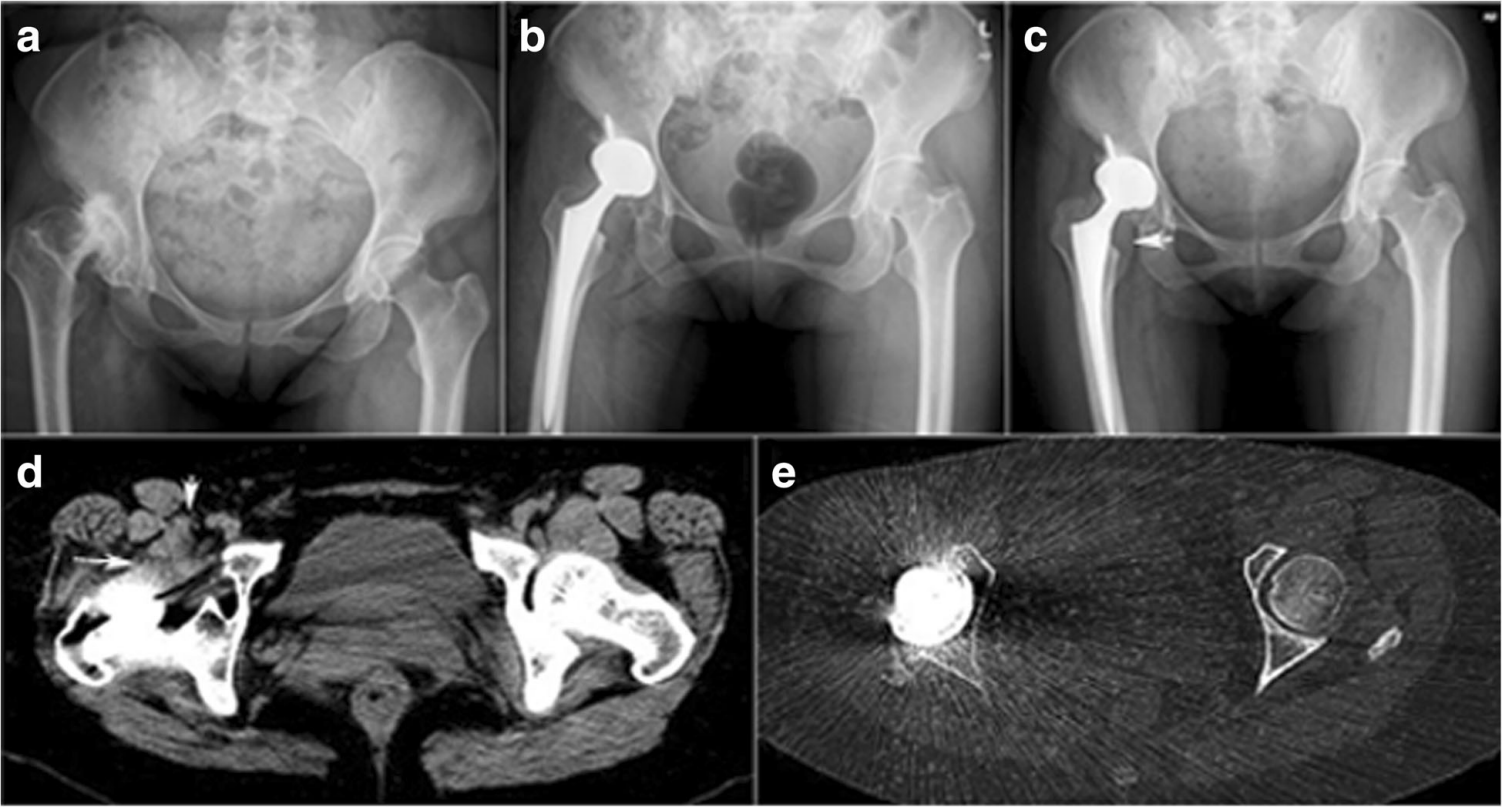
Radiographs of a 45-year-old woman are shown with right hip replacement for treating severe osteoarthritis. Dysplastic right hip with severe osteoarthritis was shown on the preoperative radiograph (**a**). Bilateral leg lengths were equal on the radiograph (**b**) at 1 month postoperatively. No complication was found until severe groin pain was complained at 5 months postoperatively. The swelling iliopsoas muscle (white arrow) was indicated on the anteroposterior radiograph (**c**) of 5 months postoperatively. **d** Computed tomographic scans of the painful hip at 5 months postoperatively. The enlarged iliopsoas (white arrow) with abnormal signal density was adhered to the thickened anterior capsule (white long arrow). **e** An over-anteverted acetabular component was detected on the computed tomographic scan at the level of acetabular component

**Fig. 2:**
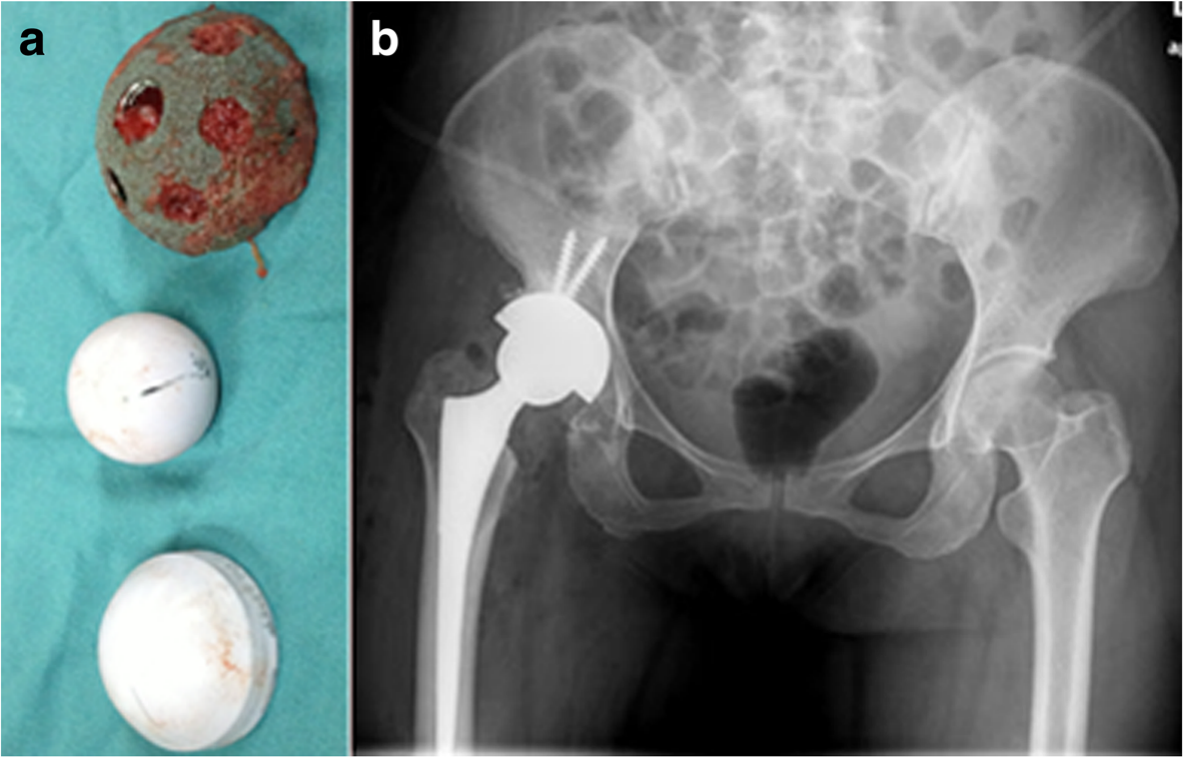
Hip revision in the foregoing patient at 6 months postoperatively. A securely fixed acetabular component was confirmed during the revision surgery. Bone ingrowth was shown within the surface of acetabular component (**a**). **b** A trabecular metal acetabular component with decreased anteversion was shown on the radiograph after hip revision

For the hip in which the peritendinous injection failed, securely fixed acetabular and femoral components appeared on postoperative AP radiographs and CT scans (Fig. 1c, e). The swelling iliopsoas muscle of the right hip was indicated on the AP radiograph and CT scans (Fig. 1c, d). Bilateral leg lengths were equal even though the dysplastic hip was 2 cm shorter preoperatively. We did not find anterior overhang of the acetabular component, protruded screws, or any other previously reported factors suggestive of iliopsoas impingement. However, the acetabular component anteversion on AP radiographs and CT scans was obviously increased (Fig. 1e) up to 43° which indicated potential anterior hip instability. Then, hip revision surgery was performed. At the time of surgery, the primary acetabular component was securely fixed with bone ingrowth (Fig. 2a) and increased anteversion. The combined cup and stem anteversion was found nearly 60°. The iliopsoas muscle was swelling and adhered to the thickened capsule. The artificial femoral head was anteriorly instable when the operative hip was extremely extended and externally rotated. Nevertheless, anterior dislocation of the artificial femoral head was prevented by the thickened capsule and swelling iliopsoas muscle. The iliopsoas tendon was released at the insertion of lesser trochanter. A 48-mm trabecular metal acetabular component with polyethylene liner (Zimmer, Warsaw, IN) was implanted with decreased anteversion (Fig. 2b). A 32-mm metal femoral head (Zimmer, Warsaw, IN) with + 3.5 mm neck length was implanted. The groin pain was completely resolved after the revision surgery. Pain-free remained at 3 years postoperatively without significant loss of hip flexion force.

Postoperative sciatic nerve palsy occurred in seven hips of the dysplastic group. Six cases of nerve palsy (3/5) disappeared in 3 months. The other case which had the maximal leg lengthening of 6.8 cm in this series improved from 2/5 to 4/5 after 1 year.

## Discussion

As a recognized cause of groin pain following total hip arthroplasty, iliopsoas tendonitis probably results from different factors. The highly dysplastic hips present reduced acetabulum depth, acetabular bone deficiency, severe leg length discrepancy, and muscular contractures. These features induced higher rates of protruded screws, leg lengthening, and anterior overhang of acetabular components following total hip arthroplasty in the dysplastic group of this study. The three factors caused iliopsoas tendonitis following hip replacement in previous reports [3, 18, 19]. However, no difference in the incidence of iliopsoas tendonitis was found between the dysplastic and control group in this study. The result was different from that of Jacobsen et al. They reported a high prevalence (56%) of iliopsoas-related pain prior to scheduled periacetabular osteotomy for Crowe type 1 dysplastic hips [13]. The preoperative iliopsoas-related pain mostly resulted from acetabular bone deficiency and hip instability which led to the higher load on soft tissue. Although the severity of hip dysplasia in our series was worse than that of Jacobsen et al, deficient femoral head coverage and hip instability were greatly treated by total hip arthroplasty. Thus, the increased load on soft tissue including iliopsoas muscle was improved after total hip arthroplasty.

The anterior overhang of acetabular components resulted from relatively retroverted or oversized acetabular cups which could lead to iliopsoas impingement. The probability of iliopsoas impingement would decrease if the cup version is anteverted over 10° compared to the native acetabular version [20]. For preventing anterior impingement and decreasing the risk of posterior dislocation, acetabular components were implanted with an anteversion of 25°–30° in all cases of this study. Both inclination and anteversion of acetabular components in the dysplastic group were similar to those of the control group. However, the anteversion was somewhat increased compared with the native acetabular version in the control group. Therefore, the anterior overhang of an acetabular component was rarely recorded in the control group (4.0%), only if the component was severely oversized. Iliopsoas tendonitis was only detected in one hip which was implanted with the maximal (56 mm) acetabular component of control group. In the dysplastic group, the original acetabulums were usually characterized by anterosuperior deficiency. The anterior overhang of acetabular components was frequently identified (30.8%) during the surgery even if the average size (44.6 mm) of acetabular components was comparably smaller than that (47.9 mm) of the control group. The acetabular component in a highly dysplastic hip was implanted with its bottom close to the inner acetabular cortex. And screws were implanted frequently for initial cup fixation and structural bulk autografts. Screw holes were drilled in different directions and measured. The length of an acetabular screw was usually designed with an increase of every 5 mm. Thus, the implanted screws sometimes were long and protruded into the pelvis. Protruded screws and leg lengthening, the other two factors of iliopsoas tendonitis, were also increased in the dysplastic group. Unexpectedly, only two cases of iliopsoas tendonitis were detected in the dysplastic group. There was a contradiction between the low rate of iliopsoas tendonitis and the increased frequency of protruded screws, leg lengthening, and anterior overhang of acetabular components in the dysplastic group. The causes of the contradiction were possibly as follows: (1) Anterior overhang of acetabular components was frequently recorded in the dysplastic group. However, the component size (38–48 mm) was relatively smaller and not big enough to irritate the iliopsoas tendon. (2) The severity of screw protrusion that induced groin pain remained poorly defined in the literature. The slightly protruded screws in this study did not perforate severely enough to induce iliopsoas tendonitis. (3) Although the leg lengthening was higher (3.6 [2.0–6.8]) in the dysplastic group, it was comparable to the preoperative LLD (3.7 [2.0–7.0]). No excessive leg lengthening was performed. (4) Iliopsoas tendon release was performed in 56 cases (42.1%) of the dysplastic group due to muscular contractures in surgeries. None was released in the control group. Since iliopsoas tenotomy has been suggested in literature as a surgical intervention for treating iliopsoas tendonitis, the iliopsoas tendonitis was somewhat prevented in the dysplastic group.

Apart from the above factors, a new cause of iliopsoas tendonitis following total hip arthroplasty was detected in a case of the dysplastic group. The iliopsoas tendonitis was irritated by an instable artificial femoral head because the acetabular component was implanted with greatly increased anteversion. This finding confirmed the previous report that iliopsoas muscle could work as an anterior stabilizer to the hip joint [13]. The iliopsoas tendon follows a pulley system that consists of the anterior border of the acetabulum, convex surface of the femoral head, and its anterior capsule [21]. After excision of the femoral head, the tendon may run in direct contact with the anterior capsule [21, 22]. The iliopsoas serves to reinforce the anterior capsule ligament as the hip is extended. The combination forces an anteverted head into internal rotation [23]. The artificial femoral head was anteriorly instable in the case. The iliopsoas tendon was repeatedly irritated by the instable artificial femoral head, whereas the irritated iliopsoas tendon and thickened capsule somewhat prevented the hip from anterior dislocation. Recently, hip instability in dysplastic hips has been reported as a cause of iliopsoas-related pain in Jacobsen’s study [13]. However, the groin pain was found prior to a surgery in Jacobsen’s study. In addition, the reported iliopsoas tendonitis following total hip arthroplasty in the literature mainly resulted from impingement against oversized acetabular or femoral components [5–10]. To our knowledge, this is the first report of iliopsoas tendonitis secondary to an instable artificial femoral head following total hip arthroplasty. The other factors of iliopsoas tendonitis were not verified in the case. The 2-cm postoperative leg lengthening was small for a dysplastic hip. Furthermore, iliopsoas tendonitis has not yet been found in our previously published series in which 21 dysplastic hips were 3.8 cm lengthened postoperatively [12]. Femoral offset was postoperatively restored in the case. The mean postoperative femoral offset of 3.4 cm in the dysplastic group was comparable to 3.4 cm in the normal Asian population [24] and 3.5 cm in the control group.

Given that the peritendinous injection failed to treat iliopsoas tendonitis in the dysplastic case, iliopsoas tendon release was considered with or without the associated acetabular component revision. A recent literature review found an overall success rate of 91.5% for surgical intervention at a mean of 22.7 months postoperatively [18, 19]. Compared to iliopsoas tenotomy, associated iliopsoas tenotomy and component revision presented similar functional outcomes but a higher rate of complications [18, 23, 25]. Single iliopsoas tenotomy could be appropriate for patients without radiographic evidences of the acetabular component malposition. Even then, it was uncertain whether iliopsoas tenotomy alone would predictably resolve the problem in this case. Potential anterior hip instability would still exist or even be exacerbated by iliopsoas tenotomy alone. The risk of postoperative hip dislocation would be higher if the reinforcement effect of iliopsoas on the anterior capsule greatly decreased after iliopsoas tenotomy. In view of the foregoing understanding, the acetabular component in the case was revised with decreased cup anteversion and iliopsoas tenotomy. Weakness in hip flexion was not found at 3 years follow-up. Because the iliopsoas tendon corresponds to the psoas muscle and the medial part of the iliac muscle, the rest of the iliac muscle is inserted on the proximal femur by fleshy fibers that are conserved in tenotomy [26].

## Limitations

This study has several limitations. Firstly, the implanted stems in the dysplastic group were greatly different from those in the control group. The Wagner Cone prosthesis was monoblock and tapered with eight longitudinal sharp ribs. The different shape of the stems possibly made a difference in our results. The collar femoral prosthesis [7] was reported as a cause of iliopsoas tendonitis in the literature. However, none of the collar femoral prosthesis was implanted in this study. Secondly, prophylactic wire fixation was used in some cases of the dysplastic group if a subtrochanteric femoral shortening osteotomy was performed. The wire cerclage was fixed beneath the lesser trochanter to avoid irritating the iliopsoas tendon. The third limitation is the relatively short duration of follow-ups in this study. Although, majority of the groin pain hips were presented within 6 months [21]. Twelve patients did not return to our clinic at the last follow-up. We considered, however, it was reasonable to include the twelve patients because they were interviewed by telephone and still satisfied with the involved hips. Finally, the iliopsoas tendonitis in the dysplastic group was possibly somewhat prevented by the iliopsoas tendon release in surgeries. Even then, no difference (*p* = 0.664) in the incidence of iliopsoas tendonitis was found between the groups excluding the cases with iliopsoas tendon release.

## Conclusions

Highly dysplastic hips did not present a higher incidence of postoperative iliopsoas tendonitis in this study. Protruded screws, leg lengthening, and anterior overhang of acetabular components really increased in the dysplastic group. However, postoperative femoral offset and inclination and anteversion of acetabular components in the dysplastic group were similar to those of the control group. Iliopsoas tendonitis was somewhat prevented by the smaller size of acetabular components and iliopsoas tendon release during the surgery of dysplastic hips. As an anterior stabilizer to the hip joint, the iliopsoas muscle in dysplastic hips possibly overloaded prior to surgeries. After total hip arthroplasty, iliopsoas tendonitis could be irritated by an artificial femoral head. Theoretically, this phenomenon could appear in other painful replaced hips which have been defined as “stable” before. On the basis of the finding in this study, we suggested that the artificial femoral head should be defined as being instable and revised even if the painful hip did not dislocate and could not be attributed to other reasons. Care should be taken when total hip arthroplasty was performed in patients with severe hip dysplasia. Totally preventing any factor related to iliopsoas tendonitis is often challenging during the surgery of these patients. Further investigation is needed to determine whether iliopsoas tendon should be prophylactically released in primary hip replacement for patients with severe hip dysplasia.

This study was a retrospective analysis of short-term results. Student’s *t* test and Pearson’s chi-square test were conducted using SPSS statistics 19 (IBM, Armonk, NY). A well-matched control group was chosen from the medical records in our institution.

## Abbreviations

AP: Anteroposterior
CT: Computed tomographic
LLD: Leg length discrepancy
